# Trends in survival of children with severe congenital heart defects by gestational age at birth: A population‐based study using administrative hospital data for England

**DOI:** 10.1111/ppe.12959

**Published:** 2023-02-06

**Authors:** Laura Gimeno, Katherine Brown, Katie Harron, Maria Peppa, Ruth Gilbert, Ruth Blackburn

**Affiliations:** ^1^ UCL Great Ormond Street Institute of Child Health London UK; ^2^ UCL Centre for Longitudinal Studies London UK; ^3^ Great Ormond Street Hospital for Children NHS Foundation Trust London UK; ^4^ UCL Institute for Health Informatics London UK

**Keywords:** administrative data, congenital heart defects, England, gestational age, survival analysis, trends

## Abstract

**Background:**

Children with congenital heart defects (CHD) are twice as likely as their peers to be born preterm (<37 weeks' gestation), yet descriptions of recent trends in long‐term survival by gestational age at birth (GA) are lacking.

**Objectives:**

To quantify changes in survival to age 5 years of children in England with severe CHD by GA.

**Methods:**

We estimated changes in survival to age five of children with severe CHD and all other children born in England between April 2004 and March 2016, overall and by GA‐group using linked hospital and mortality records.

**Results:**

Of 5,953,598 livebirths, 5.7% (339,080 of 5,953,598) were born preterm, 0.35% (20,648 of 5,953,598) died before age five and 3.6 per 1000 (21,291 of 5,953,598) had severe CHD. Adjusting for GA, under‐five mortality rates fell at a similar rate between 2004–2008 and 2012–2016 for children with severe CHD (adjusted hazard ratio [HR] 0.79, 95% CI 0.71, 0.88) and all other children (HR 0.78, 95% CI 0.76, 0.81). For children with severe CHD, overall survival to age five increased from 87.5% (95% CI 86.6, 88.4) in 2004–2008 to 89.6% (95% CI 88.9, 90.3) in 2012–2016. There was strong evidence for better survival in the ≥39‐week group (90.2%, 95% CI 89.1, 91.2 to 93%, 95% CI 92.4, 93.9), weaker evidence at 24–31 and 37–38 weeks and no evidence at 32–36 weeks. We estimate that 51 deaths (95% CI 24, 77) per year in children with severe CHD were averted in 2012–2016 compared to what would have been the case had 2004–2008 mortality rates persisted.

**Conclusions:**

Nine out of 10 children with severe CHD in 2012–2016 survived to age five. The small improvement in survival over the study period was driven by increased survival in term children. Most children with severe CHD are reaching school age and may require additional support by schools and healthcare services.

**Synopsis**

**Study question**

How has survival to age five amongst children born with severe congenital heart defects (CHD) in England changed from 2004 to 2016, overall and by gestational age at birth?

**What's already known**

During the 20th century, survival of children with CHD improved dramatically in high‐income countries but recent evidence is limited for survival to age five and how this varies by gestational age at birth.

**What this study adds**

Using a large population‐based cohort identified from linked administrative data, we show that survival to age five of children with severe CHD has continued to increase in England, driven mainly by better outcomes for children born at term.

## 1 BACKGROUND

Congenital heart defects (CHD) affect 1% of births,^1^ account for 28% of all major congenital anomalies,^2^ and are important risk factors for premature mortality.^3^ CHD is strongly associated with preterm birth (<37 weeks' gestation).^4^ Children with CHD have two times the odds of preterm birth compared to the general population.^5^ Children with CHD who were born preterm suffer a double burden of mortality and morbidity, including higher risks of long‐term chronic health conditions, neurodevelopmental and psychosocial issues,^6^, ^7^ and worse post‐surgical outcomes than children with CHD born at term.^8^, ^9^, ^10^, ^11^ CHD is also associated with more complex educational needs. In a study using linked administrative health and education data from England, children with cardiovascular conditions including CHD had a greater cumulative incidence of special education needs provision by age 11 than children without a chronic health condition, both for those born at 40 weeks (50.1% versus 28.8%) and preterm (68.0% versus 48.0% at 24–32 weeks).^12^

Survival of children born with CHD into childhood and adulthood increased dramatically in the 20th century.^13^, ^14^ Improvements in surgical and clinical management of CHD (e.g. arterial switch, Fontan and Norwood procedures, prostaglandin therapy),^15^, ^16^, ^17^ perioperative management,^18^ antenatal detection,^19^ and feeding practices have all contributed to better surgical outcomes and survival of children born with CHD.^20^ In parallel, advances in obstetric management and neonatal care have contributed to greater survival of children born preterm.^21^, ^22^ However, improvements in survival with CHD may be stalling in the 21st century. A recent Swedish study found no evidence for improvements in long‐term survival for children born after 2000.^23^

Recent evidence on trends in survival with CHD to age five in the UK is limited. Additionally, few studies have sufficient power to report recent trends in survival stratified by gestational age (GA). Disaggregating trends by GA is important since it may give insight into the mechanisms driving trends in survival. In addition, even if overall survival remains constant, increases in the survival of children born preterm with severe CHD could result in more recent cohorts reaching school age having a more complex case mix,^24^ with a greater need for educational and medical support. More accurate quantification of survival to school age (age five in England) can serve to inform families and services about the additional educational and healthcare needs of children with CHD.

In this study, we used a national birth cohort for the whole of England constructed from administrative data to describe trends over time in 5‐year survival of children with severe CHD and all other children, overall and by GA‐group.

## 2 METHODS

### 2.1 Cohort selection

We used a nationally representative birth cohort constructed from birth admissions recorded in Health Episode Statistics (HES) for England,^25^, ^26^ linked to all subsequent admissions up to age five to National Health Service (NHS) hospitals or private or charitable hospitals paid for by the NHS, and to Office for National Statistics (ONS) mortality records, using a unique pseudonymised identifier generated by NHS Digital. We followed children until their fifth birthday or death. HES captures approximately 97% of all births and 98–99% of all hospital admissions in England.^27^

The study population was restricted to singleton livebirths in England born between 1st April 2004 and 31st March 2016 recorded in HES (follow‐up to 31st March 2021). Multiple births (e.g., twins) were excluded, since they have higher mortality than singletons, higher misclassification of CHD, death and GA in administrative data (partly due to the challenge of accurately linking maternal and child records for multiple births), and are too small a group to be examined separately with sufficient statistical power.

### 2.2 Exposure

We examined the differences in 5‐year survival between three birth periods (April to March 2004–2008, 2008–2012, 2012–2016) for children with severe CHD and all other children, overall and by GA‐group (24–31, 32–36, 37–38, ≥39 weeks). Data on GA and birthweight from the mother's delivery records were used to supplement missing data in the child's birth record through the linkage of mother and baby records.^26^ We excluded children with missing GA, and those with GA <24 weeks since close to the limit of viability, there may be greater ambiguity in how cases, where the child died, are recorded (neonatal death following live birth versus miscarriage).^28^ We excluded children with implausible birthweights for GA (outside ±3 standard deviations of mean birthweight for each week of GA using growth references developed by Pan and Cole),^29^ since these may result from recording error.^26^

We restricted cases to children with severe CHD rather than any CHD as increased detection and recording of non‐severe cardiac defects over time would bias changes in prevalence and survival (Figure S1). We considered children to have severe CHD if they met at least one of the following criteria:

1. An ICD‐10 code identified by EUROCAT as indicative of severe CHD (Table S1) was recorded in any admission from birth to age five, or on the death registration.^30^ These are conditions that are likely to require surgery or to cause death, like tetralogy of Fallot, coarctation of the aorta and hypoplastic left heart syndrome (HLHS).
2. A procedural OPCS4 code indicative of cardiac surgery and therapeutic interventional catheterisation procedures (Table S2, developed in clinical consultation) was recorded in any admission up to age five, and:
  - The procedure was performed in one of 10 NHS Trusts with a paediatric cardiac centre (Table S3).^31^
  - The procedure was not a stand‐alone intervention for patent ductus arteriosus (PDA) in a child born preterm or with a birthweight <2500 g.

Our comparison group of all other children, therefore, includes children without CHD as well as those with non‐severe CHD (i.e., those with an ICD‐10 code in the range Q20‐Q26 not considered ‘severe’ by EUROCAT and who did not receive a cardiac procedure before age five, except for PDA in preterm or low birthweight children).

### 2.3 Outcomes

The outcome was all‐cause under‐five mortality. The methods used to link HES and death registrations have been described elsewhere.^32^ We identified deaths up to age five using ONS mortality records and the discharge method listed in HES admissions.

### 2.4 Statistical analysis

For children with complete data on GA, we report the number of livebirths and deaths with CHD and all other births by period and GA‐group. We also estimated the overall birth prevalence of severe CHD by GA‐group and birth period.

We explored overall differences in 5‐year survival by birth period for those with severe CHD and all other births using the Kaplan–Meier (KM) survival function. We then fitted Cox proportional hazard regression models to explore univariable associations between severe CHD, GA‐group, birth period and all‐cause under‐five mortality, and a full model to explore associations between mortality and birth period adjusting for GA and severe CHD. We tested for effect modification of the relationship between severe CHD and mortality by GA‐group and birth period, then built full models stratifying by CHD status.

We used the KM survival function to explore variation in 5‐year survival by GA group. Since the birth period may be associated with GA, and CHD is a risk factor for low GA (Figure S2), results should be interpreted carefully as stratification by GA could introduce collider bias.^33^ Using the incidence rate difference between 2004–2008 and 2012–2016 and its 95% confidence interval, we estimated the number of additional survivors with severe CHD per year in the period 2012–2016, compared to what would have been the case if 2004–2008 mortality rates had persisted, using singleton livebirth registrations between April 2012 and March 2016 (*n* = 2,594,570),^34^, ^35^ and assuming the same distribution of severe CHD and GA as in the analytical sample.

In exploratory analyses, we used the KM survival function to estimate survival to age five of children with CHD excluding deaths within 28 days of birth to evaluate whether improvements in survival persist beyond the neonatal period.

We analysed the data in Stata (version 17; StataCorp LP, College Station, TX).

### 2.5 Missing data

Gestational age is more likely to be missing for children with low birthweight or who died soon after birth (Table S4) and is likely correlated with the health of the child. The potential impact of missing data on our results was explored using multiple imputation of GA with chained equations, which is described in the Appendix S1. Owing to the paucity of available auxiliary variables and the high correlation between missing GA and missing birthweight, we report these as additional analyses.

### 2.6 Sensitivity analysis

We evaluate the impact of selection bias on our results by comparing mortality patterns (including neonatal deaths aged <28 days) and the prevalence of CHD of those included and excluded from the analytical cohort due to missing or implausible GA. We describe the proportion of children with missing GA by birth period.

We have a data‐sharing agreement with NHS Digital to use a deidentified extract of HES linked to ONS records for research on child health. We did not require research ethics approval to use these datasets.

## 3 RESULTS

**FIGURE 1:**
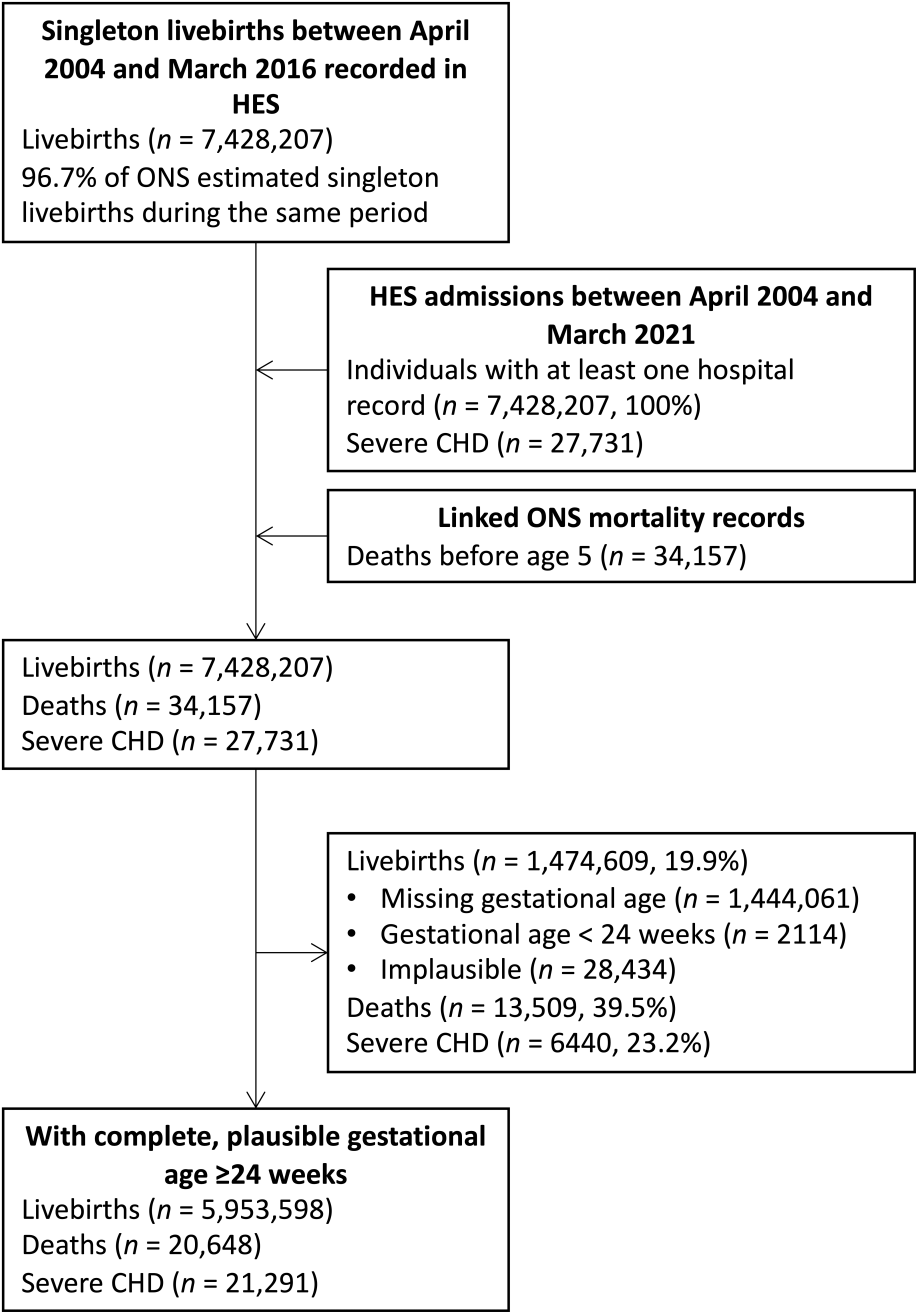
Construction of analytical cohort. We calculated the number of registered livebirths births during the study period by summing the yearly counts of livebirths in England between April 2004 and March 2016 produced by the Office for National Statistics (ONS), and by multiplying this by 0.9692 (the average proportion of singleton livebirths in England between 2012 and 2020).

Of 7,428,207 livebirths in HES and 34,157 under‐five deaths in HES and ONS data, the analytical cohort contained data on 5,953,598 (80.1%) livebirths and 20,648 (60.5%) deaths (Figure 1). Those excluded due to missing or implausible GA were more likely to die, especially soon after birth (Table S4). Due to the increasing number of births in England over time, 25.4% of births occurred in 2004–2008, 37.9% in 2008–2012 and 36.7% in 2012–2016. Approximately 5.7% of births were preterm (<37 weeks GA; Table 1). Children were more likely to have missing GA if they were born earlier in the study period (Table S4). Preterm children were over‐represented amongst deaths.

**TABLE 1:** Characteristics of singleton livebirths (*n* = 5,953,598) and deaths before age five (*n* = 20,648) in England, between 2004 and 2016.

Birth period^a^	2004–08	2008–12	2012–16	Total
	*n* (%)	*n* (%)	*n* (%)	*n* (%)
Livebirths				
Total^b^	1,512,527 (100.0%)	2,254,066 (100.0%)	2,187,005 (100.0%)	5,953,598 (100.0%)
All deaths <5 years old	5957 (0.39%)	7959 (0.33%)	6732 (0.31%)	20,648 (0.35%)
Severe CHD				
With severe CHD	4985 (0.33%)	7980 (0.35%)	8326 (0.38%)	21,291 (0.36%)
All other births	1,507,542 (99.6%)	2,246,086 (99.6%)	2,178,679 (99.6%)	5,932,307 (99.6%)
Gestational age (weeks)				
24–31	13,324 (0.9%)	18,529 (0.8%)	17,398 (0.8%)	49,251 (0.8%)
32–36	76,740 (5.1%)	106,555 (4.7%)	106,534 (4.9%)	289,829 (4.9%)
37–38	281,190 (18.6%)	403,548 (17.9%)	418,038 (19.1%)	1,102,776 (18.5%)
≥39	1,141,273 (75.5%)	1,725,434 (76.5%)	1,645,035 (75.2%)	4,511,742 (75.8%)
Deaths <5 years				
Total	5957 (100.0%)	7959 (100.0%)	6732 (100.0%)	20,648 (100.0%)
Severe CHD				
With severe CHD	621 (10.4%)	892 (11.2%)	865 (12.8%)	2378 (11.5%)
All other births	5336 (89.6%)	7067 (88.8%)	5867 (87.2%)	18,270 (88.5%)
Gestational age (weeks)				
24–31	1631 (27.4%)	2158 (27.1%)	1751 (26.0%)	5540 (26.8%)
32–36	902 (15.1%)	1188 (14.9%)	1136 (16.9%)	3226 (15.6%)
37–38	1107 (18.6%)	1479 (18.6%)	1370 (20.4%)	3956 (19.2%)
≥39	2317 (38.9%)	3134 (39.4%)	2475 (36.8%)	7926 (38.4%)

^a^ 1st April 2004–31st March 2008, 1st April 2008–31st March 2012, 1st April 2012–31st March 2016.

^b^ The smaller number of births in 2004–2008 is both the result of a higher number of excluded children due to missing gestational age, and of the smaller number of livebirths registered in England during this period.

We identified 21,291 (3.6 per 1000) children with severe CHD, of which 4978 (23%) had diagnostic codes only, 6319 (30%) had procedural codes only and 9994 (47%) had both diagnostic and procedural codes. The prevalence of severe CHD remained relatively stable over time (Table S5). Of children with severe CHD, 14.5% (3090/21,291) were born preterm, compared to 5.7% (335,990/5,932,307) of all other children. Prevalence of preterm birth remained relatively stable over time for children with severe CHD (2004–2008: 13.54%; 2012–2016: 15.69%) and all other children (2004–2008: 5.93%; 2012–2016: 5.62%). Prevalence of severe CHD was highest in the 24–31‐week group and lowest in the ≥39‐week group (Table S5). Children excluded from the analytical sample due to missing or implausible GA had a slightly higher birth prevalence of CHD (Table S4).

**FIGURE 2:**
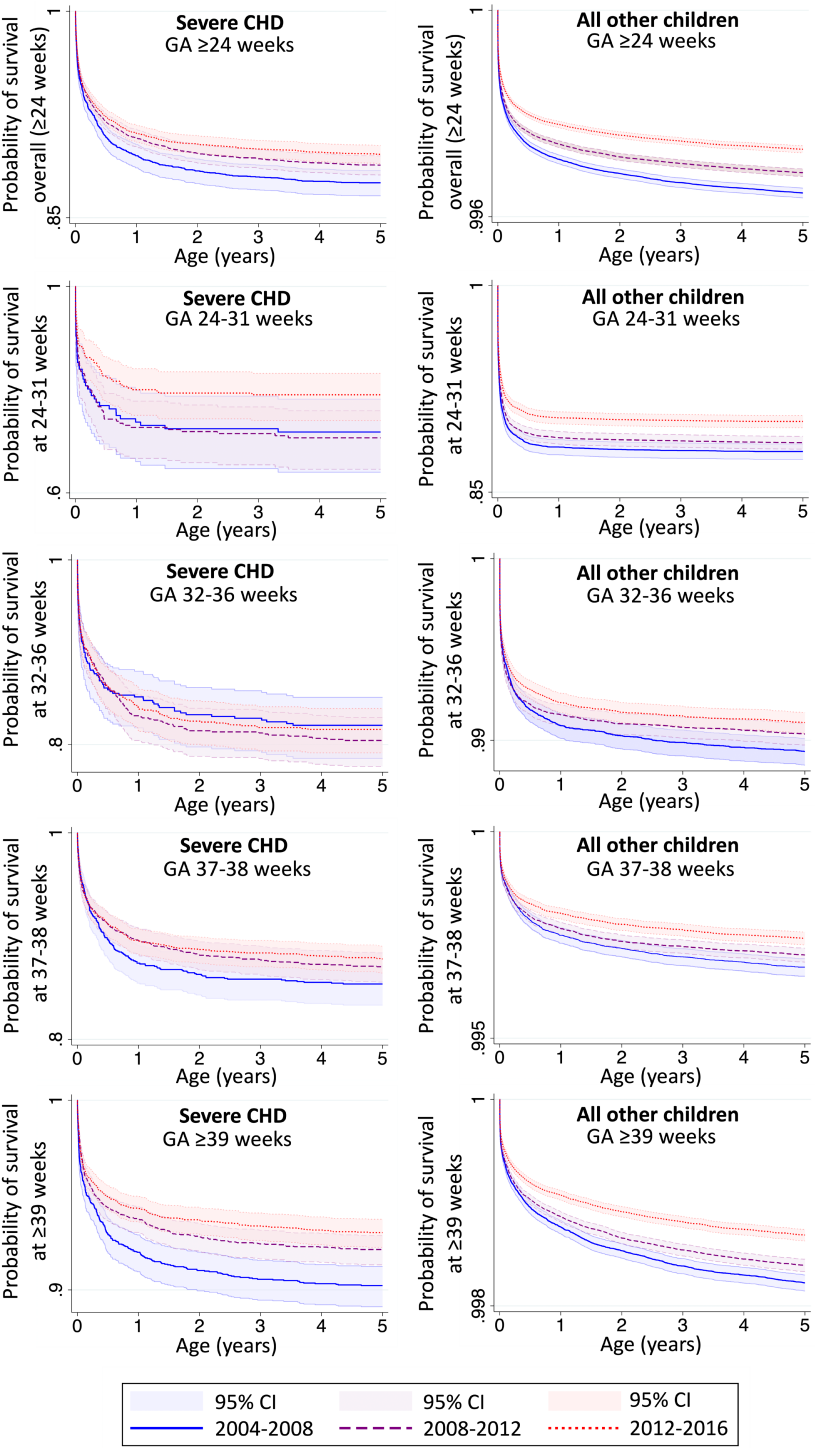
Kaplan–Meier survival plots for children with severe CHD and all other children, overall and by gestational age group.

Of all under‐five deaths, 11.5% occurred in children with severe CHD (Table 1). Survival to age five increased for children with severe CHD and all other children over the study period (Figure 2). Between 2004–2008 and 2012–2016, overall survival for children with severe CHD increased by 2.1 percentage points compared to less than 0.1 percentage points for all other children over the same period (Table 2; Figure 2).

**TABLE 2:** Percent of children born in England between 2004 and 2016 surviving to age five for children with severe CHD and all other children, by birth period and gestational age, estimated using the Kaplan–Meier survival function.

Birth period^a^	2004–2008		2008–2012		2012–2016	
Severe CHD	Yes	No	Yes	No	Yes	No
	(*n* = 4985)	(*n* = 1,507,542)	(*n* = 7980)	(*n* = 2,246,086)	(*n* = 8326)	(*n* = 2,178,679)
	% (95% CI)	% (95% CI)	% (95% CI)	% (95% CI)	% (95% CI)	% (95% CI)
Gestational age						
24–31 weeks	71.8 (64.0, 78.2)	88.0 (87.4, 88.5)	70.7 (64.6, 75.9)	88.6 (88.1, 89.1)	79.0 (74.0, 83.1)	90.1 (89.7, 90.6)
32–36 weeks	82.1 (78.5, 85.1)	98.9 (98.9, 99.0)	80.4 (77.6, 82.9)	99.0 (98.9, 99.1)	81.6 (79.1, 83.9)	99.1 (99.0, 99.2)
37–38 weeks	85.4 (83.3, 87.2)	99.7 (99.7, 99.7)	87.0 (85.5, 88.4)	99.7 (99.7, 99.7)	87.8 (86.4, 89.0)	99.7 (99.7, 99.8)
≥39 weeks	90.2 (89.1, 91.2)	99.8 (99.8, 99.8)	92.1 (91.5, 93.0)	99.8 (99.8, 99.9)	93.0 (92.4, 93.9)	99.9 (99.9, 99.9)
Overall	87.5 (86.6, 88.4)	99.7 (99.6, 99.7)	88.8 (88.1, 89.5)	99.7 (99.7, 99.7)	89.6 (88.9, 90.3)	99.7 (99.7, 99.7)
Excluded^b^	86.1 (84.9, 87.3)	99.3 (99.3, 99.3)	84.4 (82.5, 86.1)	98.9 (98.8, 99.9)	86.2 (84.4, 87.7)	99.0 (99.0, 99.0)
Overall (with excluded)	87.0 (86.2, 87.7)	99.5 (99.5, 99.5)	88.1 (87.4, 88.7)	99.6 (99.6, 99.6)	89.1 (88.4, 89.6)	99.6 (99.6, 99.7)

*Note*: Information on the number of livebirths and deaths by gestational age group and birth period underlying these results can be found in the Appendix S1 (Tables S9 and S10).

^a^ 1st April 2004–31st March 2008, 1st April 2008–31st March 2012, 1st April 2012–31st March 2016.

^b^ Excluded due to missing or implausible gestational age.

Compared to all other children, those with severe CHD had an increased risk of death before age five (Table S6). Mortality risk decreased with increasing GA and in more recent birth periods. Adjusting for CHD status and GA‐group, under‐five mortality declined by 23% between 2004–2008 and 2012–2016. There was evidence for an interaction between severe CHD and GA‐group adjusting for the birth period, but not for an interaction between severe CHD and birth period, adjusting for GA‐group. In full models stratified by CHD status (Table 3), we found strong evidence that a more recent birth period was associated with reduced mortality for those with severe CHD and for all other children. The relative reduction in mortality between 2004–2008 and 2012–2016 was greater for all other children compared to children with severe CHD but became similar after accounting for differences in GA.

**TABLE 3:** Associations of gestational age group and birth period with under‐five mortality for born children with severe CHD and all other children in England between 2004 and 2016, estimated using Cox proportional hazards regression.

	Severe CHD		All other children	
	Unadjusted HR (95% CI)^a^	Adjusted HR (95% CI)^b^	Unadjusted HR (95% CI)^a^	Adjusted HR (95% CI)^b^
Birth period^c^				
2004–2008	1.00 (Reference)	1.00 (Reference)	1.00 (Reference)	1.00 (Reference)
2008–2012	0.89 (0.80, 0.99)	0.88 (0.80, 0.98)	0.89 (0.86, 0.92)	0.92 (0.89, 0.95)
2012–2016	0.83 (0.75, 0.92)	0.79 (0.71, 0.88)	0.76 (0.73, 0.79)	0.78 (0.76, 0.81)
Gestational age				
24–31 weeks	3.62 (3.09, 4.24)	3.66 (3.12, 4.29)	76.81 (74.12, 79.60)	76.58 (74.90, 79.36)
32–36 weeks	2.49 (2.22, 2.78)	2.51 (2.24, 2.81)	6.30 (6.03, 6.58)	6.30 (6.03, 6.58)
37–38 weeks	1.67 (1.52, 1.83)	1.68 (1.53, 2.85)	1.89 (1.81, 1.97)	1.89 (1.81, 1.97)
≥39 weeks	1.00 (Reference)	1.00 (Reference)	1.00 (Reference)	1.00 (Reference)

^a^ Hazard Ratio.

^b^ All covariates in CHD status stratum‐specific models are shown in the table.

^c^ April 2004‐March 2008, April 2008‐March 2012, April 2012‐March 2016.

Stratifying by GA group, there was evidence for improved survival between 2004–2008 and 2012–2016 for children with severe CHD in the ≥39‐week group (+2.8 percentage points; Table 2; Figure 2). KM survivor function point estimates suggested that survival improved for children with CHD born at 24–31 (+7.2 percentage points) and 37–38 weeks (+2.4 percentage points). The point estimate for survival in children with severe CHD in the 32–36‐week group fell slightly (−0.5 percentage points). These patterns remained consistent after imputing missing GA (Table S7).

Applying KM survival estimates to the number of registered singleton livebirths in England between 2012 and 2016, we estimate that on average, per year between 2012 and 2016, 2214 (95% CI 2197, 2225) children with severe CHD survived to age five. This includes an additional 51 (95% CI 24, 77) children who would not have survived in 2004–2008.

After excluding neonatal deaths, we found indications of improvements in survival overall (Figure S3) and in the 37–38‐ and ≥39‐week groups (Figure S4).

## 4 COMMENT

### 4.1 Principal findings

Between 2004–2008 and 2012–2016, adjusting for GA, survival improved at a similar rate for children with severe CHD and all other children. Because of lower survival rates in children with severe CHD compared to all other children, children with severe CHD experienced a greater percentage‐point increase in survival over time. Improvements in survival for those with severe CHD varied by GA‐group, with strong evidence for better survival in the ≥39‐week group, weaker evidence in the 24–31 and 37–38‐week groups and no evidence in the 32–36‐week group.

### 4.2 Strengths of the study

The large size of the cohort achieved by using administrative data allowed us to stratify survival estimates by CHD, birth period and GA‐group. HES includes nearly 97% of births in England.^27^ We were therefore able to quantify how the analytical cohort differed from all births in England during the study period. Cases were not restricted to children who received cardiac interventions, capturing children with severe CHD who may not have received surgery because of the severity of their condition, or who died before receiving surgery because of late diagnosis.^36^ The study thus complements research using National Congenital Heart Disease Audit (NCHDA) data,^37^ which includes only children who received a cardiac procedure.

### 4.3 Limitations of the data

We relied on ICD‐10 and OPCS4 codes available in HES, which are less detailed than other coding systems like IPCCC.^38^ The coarseness of the diagnostic and procedural codes may induce some minor misclassification of CHD status, and limits in‐depth exploration of changes in case mix. Since detection and quality of recording of less severe forms of CHD has improved over time (Figure S1), we focused on severe conditions and those that require intervention, which are likely to feature in secondary care records, and for which the likelihood of detection and quality of recording in HES have likely remained similar over the study period. Whilst no studies have examined the accuracy of ICD‐10 Q‐codes in HES for detecting CHD, studies using primary care records have a high positive predictive value for CHD, above 90%.^39^ We complemented diagnostic codes with procedural codes since the latter are required for reimbursement, and so may be more complete.

We excluded a large percentage of livebirths because of missing or implausible GA. Excluded individuals were more likely to die, particularly at early ages, and had a slightly higher prevalence of CHD, so we may underestimate CHD‐related mortality. Children were more likely to have missing GA earlier in the study period. We may therefore overestimate survival primarily in 2004–2008. This could mean that improvements in survival over the study period were greater than what we observed, especially in the preterm population who we expect to feature more heavily amongst those excluded from the analytical sample. Results did however remain similar after imputing missing GA.

Restricting our study to live births rather than all pregnancies may bias our estimate of the improvement in survival upwards,^40^ particularly if advances in antenatal detection of CHD lead to an increasing proportion of severe cases ending in termination of pregnancy (TOP). Whether this is the case in England is unclear, although some evidence suggests that the TOP rate may not have increased substantially over time.^41^, ^42^ The lack of data on terminations and prenatal interventions like antenatal screening limits our ability to understand what is driving differences in survival trends by GA group.

Our analyses stratified by GA could be impacted by collider bias. We discuss this issue in greater depth in the Appendix S1, below Figure S2. As such, associations should be carefully interpreted.

### 4.4 Interpretation

An obstacle to exploring trends in survival of children with CHD by GA is the scale of data needed to be able to disaggregate by both GA and birth cohort. A large sample of children with CHD with long‐term follow‐up is required. Additionally, to evaluate the impact of changing survival on future schooling and healthcare needs, the source population from which cases are drawn should be well‐defined, which can be challenging when using hospital‐based cohorts and registry data. Much of the work on GA and CHD uses hospital‐based cohorts of children who have survived long enough to undergo surgery, and focuses on short‐term post‐surgical survival, typically until hospital discharge.^10^, ^11^, ^43^, ^44^ Few long‐term follow‐up studies exist.^45^ Population‐based and registry‐based studies have the advantage of longer follow‐up and larger sample sizes, and have been used to assess trends in survival for children with CHD overall, but most have not disaggregated by GA.^14^, ^23^, ^46^, ^47^, ^48^, ^49^ One exception is a study which used population‐based registry data from northern England to explore trends in survival to age five in children with CHD by GA from 1985 to 2003.^50^ Our work assesses trends in survival in England from 2004 onward.

In contrast to a recent Swedish study, which found no improvement in survival with CHD for children born in 2010–2017 compared to 2000–2010,^23^ we found evidence for a continued increase in the survival of children with severe CHD in 21st century England. However, we estimate that survival only increased by 2.1 percentage‐points in more than 10 years, whilst a registry‐based study in northern England found an 11.9 percentage‐point increase in 1‐year survival of children with CHD born between 1985 and 2003, from 80.7% to 92.6%.^47^

Prevalence of severe CHD in our study was slightly higher than what has been reported by others,^51^, ^52^ but this is likely due to differences in the types of CHD included in the case definition, and the inclusion or exclusion of children with chromosomal or other congenital anomalies. Our overall survival estimates to age five for children with severe CHD, between 87.5% and 89.6%, are in keeping with a pooled 5‐year survival of 85.4% (95% CI 79.4, 90.5) from a meta‐analysis by Best and Rankin.^53^

We found that overall improvements in survival with severe CHD were mainly driven by increasing survival in children born at term. Paediatric cardiac surgery teams may be less willing to intervene on children who are very preterm with complex CHD than on those born at term, because of the high risks involved. Preterm children represent 12.2% of all children receiving cardiac interventions recorded in NCHDA,^54^ but are underrepresented amongst children given surgeries for complex conditions like HLHS (2.8%),^55^ despite preterm children having 6 times higher odds of HLHS compared to children born at term.^4^ Amongst the very preterm, surgery is unlikely to be offered unless children survive long enough to put on weight, or if they have less complex CHD.

In our study, the percentage of children with CHD receiving cardiac surgery or interventional catheterisations increased with GA (Table S8). Children in the 32–36‐week group may be more likely to receive an initial cardiac intervention than those in the 24–31‐week group, even in cases where interventional treatment may not be further taken forward because of perceived high risk. More children in the 32–36‐week group may therefore be identified as cases (and contribute to mortality estimates in this group) than children in the 24–31‐week group who may die before intervention, and thus be missed as cases.

Between 2004–2008 and 2012–2016, willingness to surgically intervene in complex cases in children in the 32–36‐week group may have increased. This increased risk appetite could explain why we observed no improvement in survival for this group. Children who might not previously have been given surgery are intervened on, are, therefore, identified as cases, and contribute to deaths in this group. In contrast, the more conservative approach to surgery in the very preterm may explain the weak evidence in this study for increasing survival at 24–31 weeks. Very preterm children with a procedural code only may have simpler forms of CHD (e.g., ventricular septal defects) than those at 32–36 weeks. Cases in the 24–31‐week group may also have experienced a stronger selection effect through our restriction to livebirths and, if identified through procedural code alone, their need to survive long enough to receive surgery.

## 5 CONCLUSIONS

Survival of children with severe CHD has continued to improve in the 21st century in England, but at a slower pace than in the 20th century. This improvement is mainly the result of increasing survival amongst children with severe CHD born at term. Survival may also be improving for some preterm children with severe CHD. As such, more recent cohorts reaching school age include greater numbers of children with severe CHD, including children at the complex intersection between preterm and CHD. These children will require additional medical and educational support. Children with CHD have higher risks of chronic conditions, psychosocial and neurodevelopmental issues and poor educational outcomes compared to children without CHD. This pattern extends into adulthood, with higher risks of stroke and cardiovascular disease.^45^, ^56^ These risks are even more pronounced for children with CHD who are born preterm. Further work should assess whether greater survival in more recent cohorts translates to greater morbidity and educational needs, and how best to effectively support the needs of children with CHD in preparation for school.

## AUTHOR CONTRIBUTIONS

LG, together with RB, RG, and KH conceptualised the study, with substantial contributions from KB and MP. LG performed the statistical analyses and wrote the first draft. All authors contributed to the interpretation of results. RB, RG, KH, KB and MP read, reviewed, and critically revised the manuscript. All authors approved the final version of the manuscript.

## FUNDING INFORMATION

This project was funded through a PhD studentship awarded by the UK Medical Research Council to LG (grant number MR/N013867/1). This research was supported in part by the National Institute for Health Research through the Great Ormond Street Hospital Biomedical Research Centre and Senior Investigator funding for RG, and by Health Data Research UK (grant number LOND1), funded by the UK Medical Research Council and eight other funders. RB is supported by a UKRI Innovation Fellowship funded by the Medical Research Council (grant number MR/S003797/1). The funders had no role in the study design, data collection, data analysis, data interpretation, writing of the paper or the decision to submit the work for publication.

## CONFLICT OF INTEREST STATEMENT

The authors declare that no competing interests exist.

## Supporting information


Appendix S1.

## Data Availability

This study used NHS Hospital Episode Statistics data which was provided within the terms of data‐sharing agreement number DARS‐NIC‐393510‐D6H1D‐v7.1 to the researchers by NHS Digital. The data do not belong to the authors and may not be shared by the authors, except in aggregate form for publication. Data can be obtained by submitting a data request through the NHS Digital Data Access Request Service.
