# Supplementary material for: Trends in survival of children with severe congenital heart defects by gestational age at birth: A population‐based study using administrative hospital data for England
Source: Paediatr Perinat Epidemiol. 2023 Feb 6;37(5):390–400. doi: 10.1111/ppe.12959 (PMC10946523; doi:10.1111/ppe.12959)

SUPPLEMENTARY MATERIAL

List of tables

eTable		Page
1	EUROCAT Severe ICD-10 codes used to identify cases of severe CHD	2
2	OPCS4 procedural codes	3
3	NHS England Trusts performing paediatric congenital cardiac surgeries	4
4	Comparison of mortality patterns of those included in the analytical sample versus excluded due to missing or implausible gestational age	4
5	Prevalence of severe CHD by gestational age group and birth period (per 1,000 live births)	5
6	Crude and adjusted Hazard Ratios and 95% confidence intervals from Cox proportional hazards regression models showing association between severe CHD and under five mortality rate	5
7	Adjusted Hazard Ratios and 95% Confidence Intervals from Cox proportional hazards regression models on multiply imputed datasets showing the association between birth period and under-five mortality rate, overall and by gestational age, for children with severe CHD and all other children	6
8	Percentage of children with severe CHD with an OPCS4 code reflective of congenital cardiac surgery, by gestational age and birth period	7
9	Number of livebirths and deaths in children with severe CHD by gestational age-group and birth period	7
10	Number of livebirths and deaths in all other children by gestational age-group and birth period	7
11	Age at death (days) for children with severe CHD and all other children, and mortality record type	7
12	Birth prevalence of specific types of severe CHD per 100,000 live births, and mortality rate per 100 affected live births by diagnosis group in England 2004-2016	8
13	Distribution of timing of deaths among those with severe CHD and all other children, over time	9
14	Percent surviving to age five for children born with severe CHD and all other children, by birth period, CHD status, and gestational age, with 95% confidence intervals, estimated using the Kaplan-Meier survival function, conditional on survival to 28 days	9
15	Key references	10-12

List of figures

eFigure		Page
1	Prevalence of severe CHD, “non-severe CHD”, and “any CHD” over time (per 1,000 live births)	13
2	Directed Acyclic Graphs showing hypothesised relationships between study variables	14
3	Kaplan-Meier survival plots for children with severe CHD and all other children up to age five, conditional on survival to 28 days after birth	15
4	Kaplan-Meier survival plots for children with severe CHD and all other children by gestational age-group up to age five, conditional on survival to 28 days after birth	16
5	Kaplan-Meier survival plots for children with severe CHD and all other children up to age five, by gestational age-group	17
6	Innovations in the care and management of children born preterm and with congenital heart defects since the 1960s	17

eTable 1. EUROCAT Severe codes used to identify cases of severe CHD

ICD-10 Code	Condition
Q20.0	Common arterial trunk
Q20.1	Double outlet right ventricle
Q20.3	Discordant ventricoarterial connection
Q20.4	Double inlet ventricle
Q21.2	Atrioventricular septal defect
Q21.3	Tetralogy of Fallot
Q22.0	Pulmonary valve atresia
Q22.4	Congenital tricuspid stenosis
Q22.5	Ebstein's anomaly
Q22.6	Hypoplastic right heart syndrome
Q23.0	Congenital stenosis of aortic valve
Q23.2	Congenital mitral stenosis
Q23.3	Congenital mitral insufficiency
Q23.4	Hypoplastic left heart syndrome
Q25.1	Coarctation of the aorta
Q25.2	Atresia of the aorta
Q26.2	Total anomalous pulmonary venous connection

Reference: European Surveillance of Congenital Anomalies (EUROCAT). EUROCAT Guide 1.4: Instruction for the registration of congenital anomalies. EUROCAT Central Registry, University of Ulster; 2013 p. 93.

eTable 2. OPCS4 procedural codes

We also used OPCS4 procedural codes to identify likely instances of surgery for congenital heart disease, which served to identify cases of severe CHD where a diagnostic code was not present. These codes were developed in clinical consultation, and likely to indicate the presence of congenital heart disease if the procedures were performed before age 5.

OPCS4 Code	Condition
K196	Creation of other cardiac conduit
L021–4, L028–9	Open correction of patent ductus arteriosus
L031	Transluminal operations on abnormality of great vessel
K041–6, K048–9	Repair of tetralogy of Fallot
K051–2, K058–9	Atrial inversion operations/transposition/great arteries
K061–4, K068–9	Other repair of transposition of great arteries
K071–3, K078–9	Correction/total anomalous pulmonary venous connection
K081–4, K088–9	Repair of double outlet ventricle
K091–6, K098–9	Repair of defect of atrioventricular septum
K101–5, K108–9	Repair of defect of interatrial septum
K111–9	Repair of defect of interventricular septum
K121–5, K128–9	Repair of defect of unspecified septum of heart
K131–5, K138–9	Transluminal repair of defect of septum
K141–5, K148–9	Other open operations on septum of heart
K151–2, K158–9	Closed operations on septum of heart
K161–6, K168–9	Other therapeutic transluminal operations on septum of heart
K171–9	Repair of univentricular heart
K181–9	Creation of valved cardiac conduit
K191–5, K198–9	Creation of other cardiac conduit
K201–4, K208–9	Refashioning of atrium
K221–3, K228–9	Other operations on wall of atrium
K231–6, K238–9	
K241–9	Other operations on ventricles of heart
K251–5, K258–9	Plastic repair of mitral valve
K261–5, K268–9	Plastic repair of aortic valve
K271–6, K278–9	Plastic repair of tricuspid valve
K281–5, K288–9	Plastic repair of pulmonary valve
K291–9	Plastic repair of unspecified valve of heart
K301–5, K308–9	Revision of plastic repair of valve of heart
K311–5, K318–9	Open incision of valve of heart
K321–4, K328–9	Closed incision of valve of heart
K331–6, K338–9	Operations on aortic root
K341–6, K348–9	Other open operations on valve of heart
K351–9	Therapeutic transluminal operations on valve of heart
K361–2, K368–9	Excision of valve of heart
K371–6, K378–9	Removal/obstruction from structure adjacent/valve heart
K381–6, K388–9	Other operations on structure adjacent to valve of heart
L011–4, L018–9	Other operations for combined abnormality of great vessels
L021–4, L028–9	Open correction of patent ductus arteriosus **(only include for children with birthweight > 2500g and born at term (37 weeks gestational age or more)**
L031	Percutaneous transluminal prosthetic occlusion of patent ductus arteriosus **(only include for children with birthweight > 2500g and born at term (37 weeks gestational age or more)**
L032	Percutaneous transluminal stent implantation into arterial duct **(only include for children with birthweight > 2500g and born at term (37 weeks gestational age or more)**
L038–9	Transluminal operations on abnormality of great vessel
L041, L048–9	Open operations of the pulmonary arterial tree
L051–4, L058–9	Creation of shunt to pulmonary artery from aorta using interposition tube prosthesis
L061–9	Other connection to pulmonary artery from aorta
L071–5, L078-9	Creation of shunt to pulmonary artery from subclavian artery using interposition tube prosthesis
L081–4, L086–9	Other connection to pulmonary artery from subclavian artery
L091–2, L098–9	Other connection to pulmonary artery
L101–4, L108–9	Repair of pulmonary artery
L121–3, L126, L128–9	Other open operations on pulmonary artery
L133–6, L138-9	Transluminal operations on pulmonary artery
L231–9	Plastic repair of aorta
L691–2, L694–5, L698–9	Operations on major systemic to pulmonary artery
L801–4, L808–9	Operations on individual pulmonary veins

eTable 3. NHS England Trusts performing paediatric congenital cardiac surgeries

Hospital	Hospital Code	NHS Trust	Trust Code
Birmingham Children's Hospital	BCH	Birmingham Women's and Children's	RQ3
Bristol Royal Hospital for Children	BAC	University Hospital Bristol and Weston	RA7
Leeds General Infirmary	LGI	Leeds Teaching Hospitals	RR8
Leicester Glenfield Hospital	GRL	University Hospitals of Leicester	RWE
Liverpool Alder Hey Hospital	ACH	Alder Hey Children's	RBS
London Evelina Children's Hospital	GUY	Guy's and St Thomas'	RJ1
London Great Ormond Street	GOS	Great Ormond Street Hospital for Children	RP4
London Royal Brompton Hospital	NHB	Guy's and St Thomas'	RJ1
Newcastle Freeman Hospital	FRE	Newcastle Upon Tyne Hospitals	RTD
Southampton Wessex Cardiothoracic Centre	SGH	University Hospital Southampton	RHM

Reference: National Institute for Cardiovascular Outcomes Research (NICOR). Congenital Heart Disease in Children and Adults (Congenital Audit). CHD Procedural Activity: Centres Undergoing Major Cardiac Procedures 2017–20 [Internet]. London: NICOR; 2021. [Accessed 21st March 2022]. Available from: https://www.nicor.org/uk/congenital-heart-disease-in -children-and-adults-congenital audit/

eTable 4. Comparison of mortality patterns of those included in the analytical sample versus excluded due to missing or implausible gestational age

Livebirths					
	Analytical		Excluded		% Excluded
**Total**	5,953,598	(100.0%)	1,474,609	(100.0%)	19.9%
**Birth period**					
**2004-2008**	1,512,527	(25.4%)	849,757	(57.6)	36.0%
**2008-2012**	2,254,066	(37.9%)	312,783	(21.2)	12.2%
**2012-2016**	2,187,005	(36.7%)	312,069	(21.2)	12.5%
**Died < 5 years old**	20,648	(0.35%)	13,509	(9.2%)	39.5%
**Severe CHD**					
**Has severe CHD**	21,291	(0.36%)	6,440	(0.44%)	23.2%
**All other children**	5,932,307	(99.6%)	1,461,100	(99.6%)	19.8%
**Low birthweight (<2500g)[a]**	316,335	(5.4%)	39,366	(8.2%)	11.1%
**Deaths < 5 years**					
	Analytical		Excluded		% Excluded
**Died < 5 years old**	20,648	(100.0%)	13,509	(100.0%)	39.5%
**Severe CHD**					
**Has severe CHD**	2,378	(11.5%)	922	(6.8%)	27.9%
**All other children**	18,270	(88.5%)	12,587	(93.2%)	40.8%
**Deaths within**					
**< 48 hours**	4542	(22.0)	6752	(50.0)	59.8%
**> 48 hours & < 1 week**	2548	(12.3)	1515	(11.2)	37.3%
**> 1 week & < 1 month**	3495	(17.0)	1639	(12.1)	31.9%
**> 1 month & < 1 year**	6406	(31.0)	2492	(18.5)	28.0%
**> 1 year & < 5 years**	3657	(17.7)	1111	(8.2)	23.3%

[a]67.3% (993,126/1,474,609) of children excluded from the analytical sample had missing birthweight compared to 1.7% (100,625/5,953,598) of children included.

eTable 5. Prevalence of severe CHD by gestational age group and birth period (per 1,000 live births)

	Birth period			
	**2004-2008**	**2008-2012**	**2012-2016**	**Total**
**Gestational age**				
**24–31 weeks**	11.71 (10.16-13.68)	13.44 (11.88-15.20)	17.53 (15.68-19.59)	14.42 (13.40-15.51)
**32–36 weeks**	6.76 (6.21-7.37)	8.06 (7.54-8.62)	9.41 (88.43-10.00)	8.21 (7.89-8.55)
**37–38 weeks**	4.59 (4.35-4.85)	5.35 (5.13-5.58)	5.82 (5.59-6.05)	5.33 (5.20-5.47)
**39+ weeks**	2.65 (2.55-2.74)	2.73 (2.65-2.81)	2.79 (2.71-2.87)	2.73 (2.68-2.78)
**Total**	3.30 (3.21-3.34)	3.54 (3.46-3.62)	3.81 (3.73-3.89)	3.58 (3.53-3.62)

eTable 6. Crude and adjusted Hazard Ratios (HR) and 95% Confidence Intervals (CI) from Cox proportional hazards regression models showing association between severe CHD and under five mortality rate

	**Crude**		**Adjusted[a]**	
	**HR**	**(95% CI)**	**aHR**	**(95% CI)**
**Severe CHD**				
**All other children**	1.00	(Reference)	1.00	(Reference)
**Has severe CHD**	38.06	(36.46-39.72)	20.29	(19.42-21.19)
**Gestational age (weeks)**				
**24-31**	68.87	(66.55-71.28)	57.53	(55.55-59.58)
**32-36**	6.37	(6.11-6.63)	5.85	(5.61-6.09)
**37-38**	2.04	(1.97-2.12)	1.96	(1.89-2.04)
**≥ 39**	1.00	(Reference)	1.00	(Reference)
**Birth year**				
**2004 to 2008**	1.00	(Reference)	1.00	(Reference)
**2008 to 2012**	0.90	(0.87-0.93)	0.91	(0.88-0.94)
**2012 to 2016**	0.78	(0.75-0.81)	0.77	(0.75-0.80)

[a]All covariates in the model are shown in this table (severe CHD status, gestational age-group, and birth period)

eTable 7 Adjusted Hazard Ratios (aHR) and 95% Confidence Intervals (CI) from Cox proportional hazards regression models on multiply imputed datasets showing the association between birth period and under-five mortality rate, overall and by gestational age, for children with severe CHD and all other children

	2004-2008	2008-2012	2012-2016
	aHR (95% CI)	aHR (95% CI)	aHR (95% CI)
With severe CHD[a]	1.00 (Ref)	0.84 (0.77-0.91)	0.76 (0.69-0.83)
With severe CHD & 24-31 weeks	1.00 (Ref)	0.94 (0.64-1.38)	0.69 (0.48-1.02)
With severe CHD & 32-36 weeks	1.00 (Ref)	1.05 (0.81-1.36)	0.99 (0.77-1.29)
With severe CHD & 37-38 weeks	1.00 (Ref)	0.85 (0.71-1.03)	0.80 (0.67-0.97)
With severe CHD & 39+ weeks	1.00 (Ref)	0.79 (0.71-0.88)	0.71 (0.63-0.79)
All other children[a]	1.00 (Ref)	0.79 (0.77-0.81)	0.68 (0.66-0.70)
All other children & 24-31 weeks	1.00 (Ref)	0.94 (0.88-1.01)	0.81 (0.76-0.88)
All other children & 32-36 weeks	1.00 (Ref)	0.90 (0.81-0.98)	0.83 (0.76-0.92)
All other children & 37-38 weeks	1.00 (Ref)	0.91 (0.84-0.98)	0.78 (0.71-0.85)
All other children & 39+ weeks	1.00 (Ref)	0.72 (0.70-0.75)	0.61 (0.59-0.64)

[a] Controlling for gestational age-group

Exploring the impact of missing data on gestational age using multiple imputation

We found that GA is more likely to be missing for children with low birthweight or who died soon after birth (eTable 4), and is likely correlated with the health of the child. Since missingness is correlated with unobservable variables, it is therefore likely to be missing not at random. To explore the impact of selection bias arising from missing data on GA on our results, we used multiple imputation to estimate missing values of GA. We note that multiple imputation is valid under the assumption of data being missing at random (which is likely not met in this analysis). However, we include it for consideration alongside the complete case analysis.

Variables included in the imputation model were death before age 5 (assumed to be observed for all individuals), severe CHD status (assumed to be observed for all individuals), birth period (observed for all individuals), GA (19.4% missing), birthweight (14.7% missing), maternal age (2.8% missing), and sex (0.15% missing). GA, birthweight, and maternal age were imputed as continuous variables, and sex as a binary variable. Maternal age was included as an auxiliary variable. Birthweight and sex were included both as auxiliary variables and because they are used to identify implausible combinations of GA and birthweight in each imputed dataset, meaning that we were able to apply the same exclusion criteria as in our complete case analysis. After imputing GA as continuous, we regrouped it into the same categories as those used in the complete case analysis (24-31, 32-35, 37-38 and ≥39 weeks).

Our imputation model imputed GA, birthweight, sex, and maternal age separately by CHD status and birth period. To support survival analysis using imputed data, we included the Nelson-Aalen estimate of the cumulative hazard function in the imputation model. Using chained equations, we generated 25 imputed datasets (m = 25). The fraction of missing information (FMI) was ~20%, so this choice of m satisfies a rule of thumb developed by White and colleagues,[1] where m should be chosen such that FMI/m ≈ 0.001. Given the FMI, m = 25 is likely to yield efficiencies close to that of m = 100.[2]

[1] White IR, Royston P & Wood AM. Multiple imputation using chained equations: Issues and guidance for practice. *Stat Med.* 2011;**30**(4):377-399

[2] Graham JW, Olchowski AE & Gildreath TD. How many imputations are really needed? Some practical clarifications of multiple imputation theory. *Prev Sci.* 2007;**8**:206-213

eTable 8. Percentage of children with severe CHD with an OPCS4 code reflective of congenital cardiac surgery, by gestational age and birth period

	2004-2008		2008-2012		2012-2016		Total	
	N	(%)	N	(%)	N	(%)	N	(%)
**24-31 weeks**	86	(55.1)	107	(43.0)	139	(45.6)	332	(46.8)
**32-36 weeks**	364	(70.1)	569	(66.1)	672	(67.1)	1605	(67.4)
**37-38 weeks**	1009	(78.2)	1660	(76.9)	1871	(76.9)	4540	(77.2)
**39+ weeks**	2397	(79.4)	3793	(80.5)	3646	(79.5)	9836	(79.8)
**Total**	3856	(77.4)	6129	(76.8)	6328	(76.0)	16313	(76.6)

eTable 9. Number of livebirths and deaths before age five in children with severe CHD by gestational age-group and birth period. These are the counts that underlie the percentages surviving by birth period, severe CHD status and gestational age reported in Table 2 in the main manuscript.

	2004-2008		2008-2012		2012-2016		Total	
**Gestational age**	**Livebirths**	**Deaths**	**Livebirths**	**Deaths**	**Livebirths**	**Deaths**	**Livebirths**	**Deaths**
**24-31**	156	44	249	73	305	64	710	181
**32-36**	519	93	859	168	1,002	184	2,380	445
**37-38**	1,291	189	2,159	280	2,432	297	5,882	766
**39+**	3,019	295	4,713	371	4,587	320	12,319	986
**Total**	4,985	621	7,980	892	8,326	865	21,291	2,378

eTable 10. Number of livebirths and deaths before age five in all other children by gestational age-group and birth period. These are the counts that underlie the percentages surviving by birth period, severe CHD status and gestational age reported in Table 2 in the main manuscript.

	2004-2008		2008-2012		2012-2016		Total	
**Gestational age**	**Livebirths**	**Deaths**	**Livebirths**	**Deaths**	**Livebirths**	**Deaths**	**Livebirths**	**Deaths**
**24-31**	13,168	1,587	18,280	2,085	17,093	1,687	48,541	5,359
**32-36**	76,221	809	105,696	1,020	105,532	952	287,449	2,781
**37-38**	279,899	918	401,289	1,199	415,606	1,073	1,096,894	3,190
**39+**	1,138,254	2,022	1,720,721	2,763	1,640,448	2,155	4,499,423	6,940
**Total**	1,507,542	5,336	2,246,086	7,067	2,178,679	5,867	5,932,307	18,270

eTable 11. Age at death (days) for children with severe CHD and all other children, and mortality record type

	Mean	(SD)	N
**Total**	202	(373)	20,648
**Severe CHD**			
**Yes**	188	(319)	2,378
**No**	204	(380)	18,270
**Record Type**			
**ONS only**	339	(440)	6,750
**HES only**	41	(200)	708
**Both**	141	(319)	13,190

eTable 12. Birth prevalence of specific types of severe CHD per 100,000 live births, and mortality rate per 100 affected live births by diagnosis group in England 2004-2016

	Livebirths		Deaths	
	N	Prevalence[a]	N	Mortality rate[b]
**CAT**	361	6.06	92	25.5
**DORV**	1,167	19.6	230	19.7
**TGA**	2,317	38.9	274	11.8
**DIV**	356	6.0	65	18.3
**AVSD**	3,449	57.9	581	16.8
**Fallot**	2,712	45.6	250	9.2
**PVA**	779	13.1	173	22.2
**CTS**	415	7.0	96	23.1
**Ebstein**	322	5.4	66	20.5
**HRHS**	333	5.6	81	24.3
**CSAV**	1,260	21.2	195	15.5
**CMS**	427	7.1	106	24.8
**CMI**	1,716	28.8	201	11.7
**HLHS**	1,347	22.6	580	43.1
**COA**	3,189	53.6	408	12.8
**AA**	201	3.4	53	26.4
**TAPVC**	567	9.5	127	22.4

[a] Prevalence per 100,000 live births
[b] Mortality rate per 100 affected cases

CTA = common arterial trunk; DORV = double outlet right ventricle; TGA = transposition of the great arteries; DIV = double inlet ventricle; AVSD = atrioventricular septal defect; Fallot = tetralogy of Fallot; PVA = pulmonary valve atresia; CTS = congenital tricuspid stenosis; Ebstein = Ebstein's anomaly; HRHS = hypoplastic right heart syndrome; CSAV = congenital stenosis of aortic valve; CMS = congenital mitral stenosis; CMI = congenital mitral insufficiency; HLHS = hypoplastic left heart syndrome; COA = coarctation of the aorta, AA = atresia of the aorta; TAPVC = total anomalous pulmonary venous connection.

Note: Counts do not add up to the total number with severe CHD, as some individuals have multiple diagnoses, and these cases are identified using ICD-10 codes in HES and mortality records only (i.e., no procedural codes used).

eTable 13. Distribution of timing of deaths among those with severe CHD and all other children, over time

	2004–2008				2008–2012				2012–2016				Total			
**Severe CHD**	**Yes**	**(%)**	**No**	**(%)**	**Yes**	**(%)**	**No**	**(%)**	**Yes**	**(%)**	**No**	**(%)**	**Yes**	**(%)**	**No**	**(%)**
**Deaths within**																
**< 48 hours**	48	(7.7)	1223	(22.9)	88	(9.9)	1707	(24.2)	80	(9.2)	1396	(23.8)	216	(9.1)	4326	(23.7)
**> 48 hours & < 1 week**	75	(12.1)	631	(11.8)	94	(10.5)	912	(12.9)	93	(10.8)	743	(12.7)	262	(11.0)	2286	(12.5)
**> 1 week & < 1 month**	137	(22.1)	839	(15.7)	202	(22.6)	1106	(15.7)	225	(26.0)	986	(16.8)	564	(23.7)	2931	(16.0)
**> 1 month & < 1 year**	263	(42.4)	1660	(31.1)	348	(39.0)	2101	(29.7)	340	(39.3)	1694	(28.9)	951	(40.0)	5455	(30.0)
**> 1 year & < 5 years**	98	(15.8)	983	(18.4)	160	(17.9)	1241	(17.6)	127	(14.7)	1048	(17.9)	385	(16.2)	3272	(17.9)
**Total < 5 years**	621	(100.0)	5336	(100.0)	892	(100.0)	7067	(100.0)	865	(100.0)	5867	(100.0)	2378	(100.0)	18270	(100.0)

eTable 14. Percent surviving to age five for children born with severe CHD and all other children, by birth period and gestational age, with 95% confidence intervals (CI), estimated using the Kaplan-Meier survival function, conditional on survival to 28 days

	2004-2008				2008-2012				2012-2016			
**Severe CHD**	**Yes**		**No**		**Yes**		**No**		**Yes**		**No**	
	**Perc**	**(95% CI)**	**Perc**	**(95% CI)**	**Perc**	**(95% CI)**	**Perc**	**(95% CI)**	**Perc**	**(95% CI)**	**Perc**	**(95% CI)**
**Gestational age**												
**24-31 weeks**	85.5	(78.2-90.5)	96.7	(96.4-97.0)	84.2	(78.5-88.5)	96.7	(96.4-97.0)	89.3	(84.9-92.4)	97.2	(96.9-97.4)
**32-36 weeks**	90.1	(87.0-92.4)	99.5	(99.4-99.5)	87.9	(85.4-90.0)	99.6	(99.6-99.7)	89.0	(86.8-90.9)	99.6	(99.6-99.6)
**37-38 weeks**	89.7	(87.8-91.2)	99.8	(99.8-99.8)	91.8	(90.5-92.9)	99.8	(99.8-99.8)	92.3	(91.1-93.3)	99.9	(99.8-99.9)
**39+ weeks**	93.9	(93.0-94.7)	99.9	(99.9-99.9)	95.1	(94.5-95.8)	99.9	(99.9-99.9)	96.1	(95.5-96.7)	99.9	(99.9-99.9)
**Overall**	92.0	(91.3-92.5)	99.8	(99.8-99.8)	92.8	(92.2-93.3)	99.8	(99.8-99.8)	93.7	(93.2-94.2)	99.9	(99.9-99.9)

eTable 15. Key references

Reference	Study population	Exposures	Outcome	Results
Costello *et al.* (2014)	**Hospital-based** cohort from the US. Neonates who underwent a primary cardiovascular operation before 28 days of age between 2010 and 2011. N = 4,784	Full-term (39+ weeks) versus early term (37-38 weeks)	In-hospital mortality	In-hospital mortality was 9.9% overall, 7.3% for 39-40 weeks, 9.0% at 38 weeks, and 13.2% at 37 weeks.
Cheung *et al.* (2021)	**Hospital-based** cohort from Canada. All preterm (<37 weeks) children receiving open heart surgery within 6 weeks of birth between 1996 and 2016, N = 115	Gestational age < 37 weeks, complex congenital heart defects	Deaths before first discharge, and before 2 years of age	10% died before first discharge, 18% died before 2 years of age
Costello *et al.* (2010)	**Hospital-based** cohort from the US. Neonates with critical CHD and known gestational age in cardiac ICU at Children's Hospital Boston between 2002 and 2008. N = 971<br>Critical congenital heart defects are defined as those requiring surgical or transcatheter cardiac intervention or resulting in death < 28 days. Exclude those with chromosomal anomalies and other major malformations.	Gestational age (37-38, 39-40 and 41+ weeks)	In-hospital mortality	6.9% mortality in 37-38-week group, 2.6% in 39-40-week group, and 8% for 41+ weeks
Knowles *et al.* (2014)	**Multicentre hospital cohort** of children diagnosed with severe CHD before age 1 born between 1992 and 1995 in England and Scotland (UK Collaborative Study of Congenital Heart Defects), linked to mortality registry. N = 3,897	Serious congenital heart defects are defined as those cases requiring surgical intervention or resulting in death before age 1.	Survival to one year of age and to 15 years of age	1-year survival was 79.8% and 15-year survival was 71.1%. 78% of deaths occurred in the first year of life.
Savorgnan *et al.* (2021)	**Multicentre register of paediatric cardiac surgery** from the US (Pediatric Cardiac Critical Care Consortium database). Neonates born at 34-41 weeks gestational age, who underwent cardiac surgery before the end of the neonatal period (44 weeks), and were hospitalised between 1st August 2014 and 2nd January 2019. N = 2,298. Exclude operations within first 24 hours of life. Included procedures are Norwood procedure, hybrid stage 1 palliation procedure, benchmark biventricular repairs (including arterial switch, arterial switch with ventricular septal defect closure, truncus arteriosus repair, and tetralogy of Fallot)	Gestational age at birth (late preterm: 34-36 weeks, early term: 37-38 weeks, full-term: 39-40 weeks, late term: 41 weeks)	In-hospital mortality	Increased odds of mortality for preterm compared to full-term, but no difference for early term versus full-term.
Spector *et al.* (2018)	**Multicentre register of paediatric cardiac surgery** from the US (Pediatric Cardiac Care Consortium database). Any patient who received first congenital heart surgery < 21 years of age between January 1982 and April 2003, and with identifiers allowing linkage to National Death Index through to 2014. N = 35,998. Exclude isolated ductal ligation in preterm infants weighing less than 2.5 kg	Congenital heart defects classified in different levels of severity (mild, moderate, severe) depending on diagnosis and operating strategy.	Overall standardised mortality rate (SMR), 15-year SMR	Overall SMR was 8.3. 15-year SMR decreased from 12.7 in 1982-1992 to 10 in 1998-2003.

eTable 15 (continued)

Best & Rankin (2014)	Systematic review of 16 studies which ascertain all people born with CHD in a predefined area, with follow-up beginning from birth. **Almost all studies are registry-based.**	Congenital heart defects. Heterogeneity in the criteria used to define cases across studies.	Survival to at least 5 years of age	Pooled 1-year survival was 87%, pooled 5-year survival was 85.4% and pooled 10-year survival was 81.4%. Heterogeneity of estimates between articles.
Dadvand *et al.* (2009)	**Registry-based** study from northern England (NorCAS), 1985 to 2003. Covers the counties of Tyne and Wear, Cleveland, Durham, Northumberland, and North Cumbria. Include late miscarriage, livebirths, stillbirths, terminations of pregnancy for foetal anomaly. Allow both prenatal and postnatal diagnosis.	Congenital heart defects (Q20-Q26) using EUROCAT minor exclusions list (i.e., excludes functional/unspecified heart murmur, patent ductus arteriosus associated with prematurity, peripheral pulmonary artery stenosis, and congenital heart block)	Survival to one year of age	Overall survival to age 1 was 89.2% over the study period, increasing from 80.7% in 1985 to 92.6% in 2003.
Oster *et al.* (2013)	**Registry-based** study from Atlanta metro area, USA (Metropolitan Atlanta Congenital Defects Program). Children born between 1979 to 2005 and with CHD diagnosed before age 6. N = 6,965 of which 1,830 with critical CHD	Critical congenital heart defects (12 conditions: hypoplastic left heart syndrome, pulmonary atresia, tetralogy of Fallot, transposition of the great arteries, tricuspid atresia, truncus arteriosus, total anomalous pulmonary venous return, coarctation of the aorta, double outlet right ventricle, Ebstein anomaly, interrupted aortic arch, singe ventricle), without noncardiac defects or chromosomal disorders.	Survival to one year of age	Survival to age 1 was 75.2% for those with critical CHD, and 97.1% for non-critical CHD. Improved survival for those with critical CHD from 67.4% in 1979-1993 to 82.5% in 1994-2005.
Best *et al.* (2017)	**Registry-based** study in northern England (NorCAS), live births between 1985 and 2008. N = 5070. Cases of CHD identified using ICD-10 codes Q20-Q26. Exclude EUROCAT minor conditions, such as isolated patent ductus arteriosus in preterm infants.	Year of birth, gestational age (< 32, 32-36, 37-41, ≥42 weeks), birthweight (low/average/ high), extracardiac anomalies (none, structural, chromosomal/genetic), maternal age, sex, deprivation (most/average/least), severity (I, II, III, unclassified)	Survival to 5 years of age	Gestational age, birthweight and year of birth are significant predictors of mortality. In 2003, children born at term at normal birthweight had a 96.3% chance of surviving to age 5.
Coi *et al.* (2022)	**Registry-based** study from countries in Western Europe (13 EUROCAT surveillance centres in Denmark, Finland, France, Italy, Malta, the Netherlands, Norway, Spain, and the UK), for livebirths between 1995 and 2014 with rare congenital structural anomalies. N = 12,685	Focusing on rare structural congenital anomalies (prevalence < 1 per 10,000). For CHD, focus on common arterial truncus, double outlet right ventricle, single ventricle, tricuspid atresia and stenosis, Ebstein anomaly, pulmonary valve atresia, hypoplastic right heart, aortic atresia/interrupted aortic arch, and total anomalous venous return.	Survival to 1 week, 4 weeks, 1 year, 5 years, and 10 years of age	Percentage survival to age 5: 61.4% for common arterial truncus, 79.5% for double outlet right ventricle, 72.1% for single ventricle, 77.9% for tricuspid atresia and stenosis, 78.9% for Ebstein's anomaly, 76.2% for pulmonary valve atresia, 72.7% for hypoplastic right heart, 63.0% for aortic atresia/interrupted aortic arch, and 76.4% for total anomalous venous return.

eTable 15 (continued)

Khairy *et al.* (2010)	**Population-based** study in Quebec, Canada, using linked hospital and death registration data. Patients with at least one diagnostic ICD-9 code for CHD and/or surgical procedure specific to these codes in their hospital records between January 1983 and June 2005, alive or born after July 1987. N = 982,363	Congenital heart defects based on ICD-9 codes in hospital admission records. Classified into five blocks based on severity.	Deaths before June 2005	31% decrease in mortality between 1987-1990 and 2002-2005. For adults aged 18 to 64, reductions in mortality parallel general population, but faster reductions among children.
Steurer *et al.* (2017)	**Population-based** study from California, USA. All livebirths in California between 2005 and 2012 using linked hospital discharge and readmission data, birth records and death records.	Critical congenital heart defects excluding chromosomal anomalies using ICD-9 codes in birth, transfer and readmission records. Critical congenital heart defects defined as CHD that is "likely to be detected by pulse oximetry screening some or most of the time". Primary targets for screening are: hypoplastic left heart syndrome, pulmonary atresia, tetralogy of Fallot, transposition of the great arteries, tricuspid atresia, truncus arteriosus. Secondary targets are coarctation of the aorta, double outlet right ventricle, Ebstein anomaly, sand single ventricle. Also include pulmonary and aortic stenosis requiring intervention in the first two years of life. Gestational age group (< 29, 29-31, 32-34, 35-36, 37-38, and 39-42 weeks)	Death before one year of age	18% of children with critical CHD born premature. 57.9% of children died before age 1 in the < 29 weeks group, compared 0.1% in the 39-42-week group
Mandalenakis *et al.* (2020)	**Population-based** study in Sweden, linked inpatient hospital data and mortality registry data, for births between 1980 and 2017. 64,396 with CHD, matched to 630,012 controls without CHD. Followed to 2017.	Congenital heart defects diagnosed at any time during the study. Defined as patients with at least one hospital discharge or death certificate with ICD-8, ICD-9 or ICD-10 code indicative of CHD. Also categorised into different lesion groups.	Survival up to 2017	Almost all deaths occur before age 4. No change in survival for those born in 2010-2017 compared to those born in 2000-2010. Estimate that 95% of those born with any CHD in 21st century will survive to adulthood.

eFigure 1. Prevalence of severe CHD, "non-severe CHD", and "any CHD" over time (per 1,000 live births)

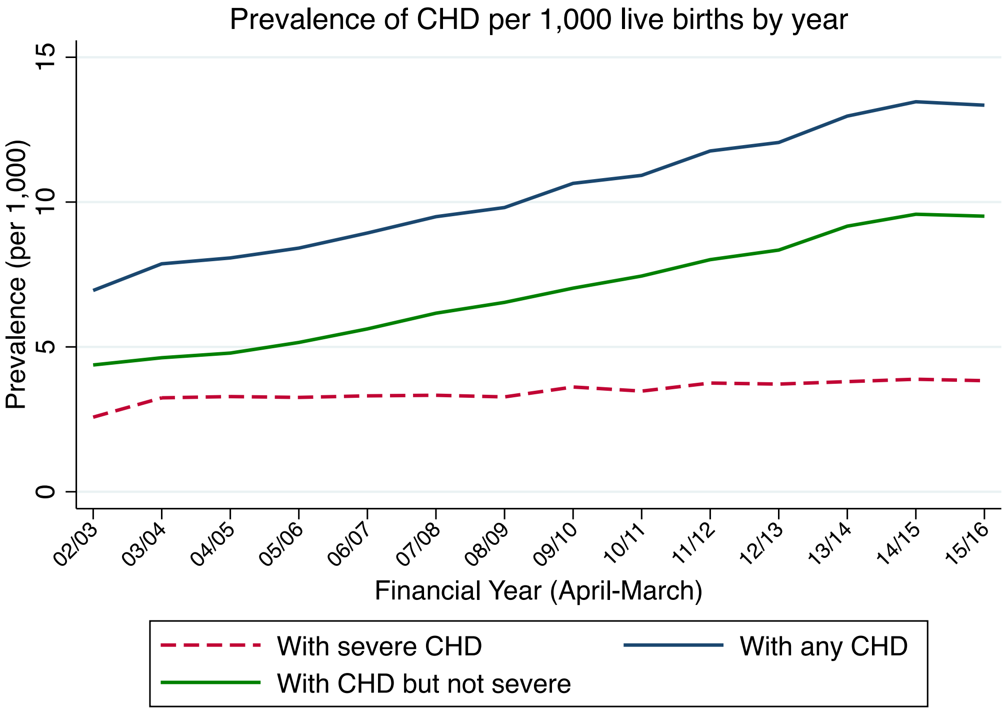

The coding depth in HES means that it is not possible to narrow down "any CHD" to take into account some exclusions made by EUROCAT (e.g., patent foramen ovale, which is considered minor), so the category "any CHD" corresponds to ICD-10 codes Q20 to Q26. Those with "non-severe CHD" correspond to the difference between the group "any CHD" and "severe CHD". We can see that the increase in the prevalence of "any CHD" is driven by an increase in diagnosis codes outside the EUROCAT severe code-list. This is likely to be driven by improvements in the quality of the records, and by better antenatal detection of cases of CHD which may not have been picked up in earlier periods.

This also informed the choice of our comparison group in our regression analyses. Detection of "non-severe" CHD is likely to have increased over time. This means that the subset of children with milder heart defects (requiring less intensive management and low risk of death) will be larger in 2012-16 than for earlier time periods, thus introducing a time-dependent ascertainment bias if a comparison group of "no CHD" is used. For this reason, we chose to compare those with severe CHD to all those without severe CHD (a group which includes those with less severe types of CHD), as we believe this to be a more uniform comparison group over time.

The crude risk of dying before age 5 for those with no CHD across the entire period of study was 2.55 per 1,000, compared to 3.06 per 1,000 for those with no severe CHD. For comparative purposes, crude mortality rate before age 5 for those with severe CHD was 110.01 per 1,000.

eFigure 2. Directed Acyclic Graphs showing hypothesised relationships between study variables

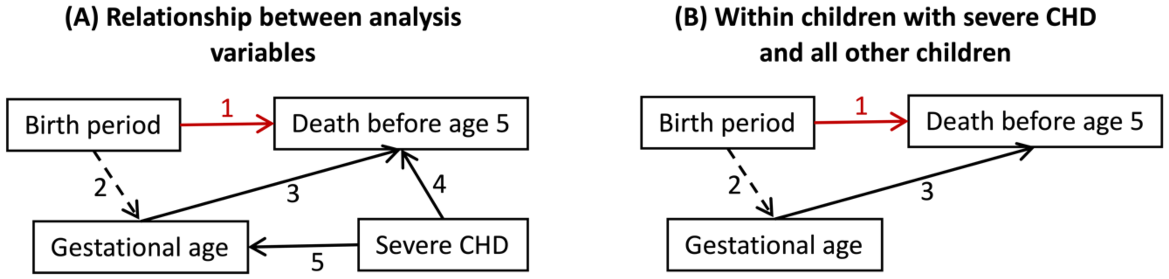

In panel A, we show the relationship between all variables considered in this study, and in panel B we show how we conceptualised relationships between variables within the two strata: children with severe CHD and all other children. The relationships that we describe in these diagrams are as follows:

1. The association between birth period and death before age 5 is the focus of this study. Has survival improved over time? That is, is later birth period associated with lower risk of death before age 5?
2. We could conceive of a relationship between GA and birth period. GA cannot affect birth period. However, as scanning and medical intervention have changed (and generally increased) over time, it is possible that there are more liveborn preterm children in later birth periods. This relationship was not strongly apparent when we explored the data (% born preterm remained relatively stable for children with CHD and all other children), so we have represented this relationship with a dashed line.
3. Low GA is a risk factor for death before age 5.
4. Severe CHD is a risk factor for death before age 5.
5. Severe CHD is a risk factor for preterm birth (low GA). There is one situation in which it could be said that low GA causes CHD: isolated patent ductus arteriosus in children born preterm or with a low birthweight. Our definition of severe CHD excludes these children, so this arrow must flow from CHD to GA.

Whether stratifying on or adjusting for GA is problematic for estimating trends in survival over time for children with severe CHD and all other children hinges on the nature of relationship 2 in the DAGs above. That is, whether GA is an ‘effect’ of birth period, since this determines whether GA is on the causal pathway between birth period and death, and whether GA is a collider when stratifying by GA and comparing trends children with severe CHD and all other children.

In our Cox regression models (Table 3), we show that within each stratum (all other children and children with CHD) adjusting for GA does not strongly impact effect estimates for birth period compared to unadjusted models, and does not change our conclusion that survival has improved for both groups over the period. This suggests, depending on how we conceptualise the relationship between birth period and GA, that the relationship between birth period and death is only weakly confounded by GA, or that only a very small part of the total effect of birth period on mortality within strata is explained by birth period’s effect on GA. In our Kaplan-Meier analyses for all births ≥24 weeks GA, we do not adjust for GA (and this is what our calculation for the number of deaths averted and our conclusion that survival has continued to increase for children with CHD are based on).

The aim of this paper was to evaluate whether there was evidence for improving survival over time for children with severe CHD, and to describe trends by age-group. We present results for all other children to help the reader to contextualise trends for children with CHD. Direct comparisons between trends for children with severe CHD and all other children could be biased if stratification by GA introduces collider bias. Whether this is the case is unclear as it depends on whether birth period affects GA, and we did not find strong evidence for this in our data. However, as there is a possibility that stratifying on both CHD and GA induces some collider bias, we suggest that readers should interpret results, and specifically the differences between trends for children with severe CHD and all other children, with caution.

We nevertheless believe that this hypothesis-generating work exploring changing survival over time by GA in England can provide valuable information to clinicians and researchers, given innovations in the care of preterm children and children with CHD in the 21st century, and to explore how changes in survival affect the composition of cohorts of children surviving to school age.

eFigure 3. Kaplan-Meier survival plots for children with severe CHD and all other children up to age five, conditional on survival to 28 days after birth

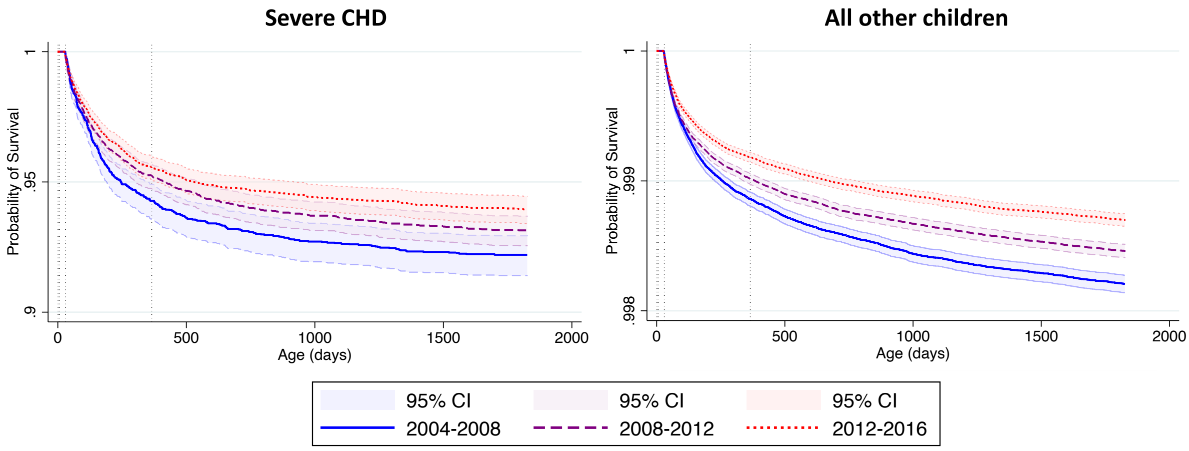

Dashes indicate 48 hours after birth, 1 week after birth, 1 month after birth, and 1 year after birth.

eFigure 4. Kaplan-Meier survival plots for children with severe CHD and all other children by gestational age-group up to age five, conditional on survival to 28 days after birth

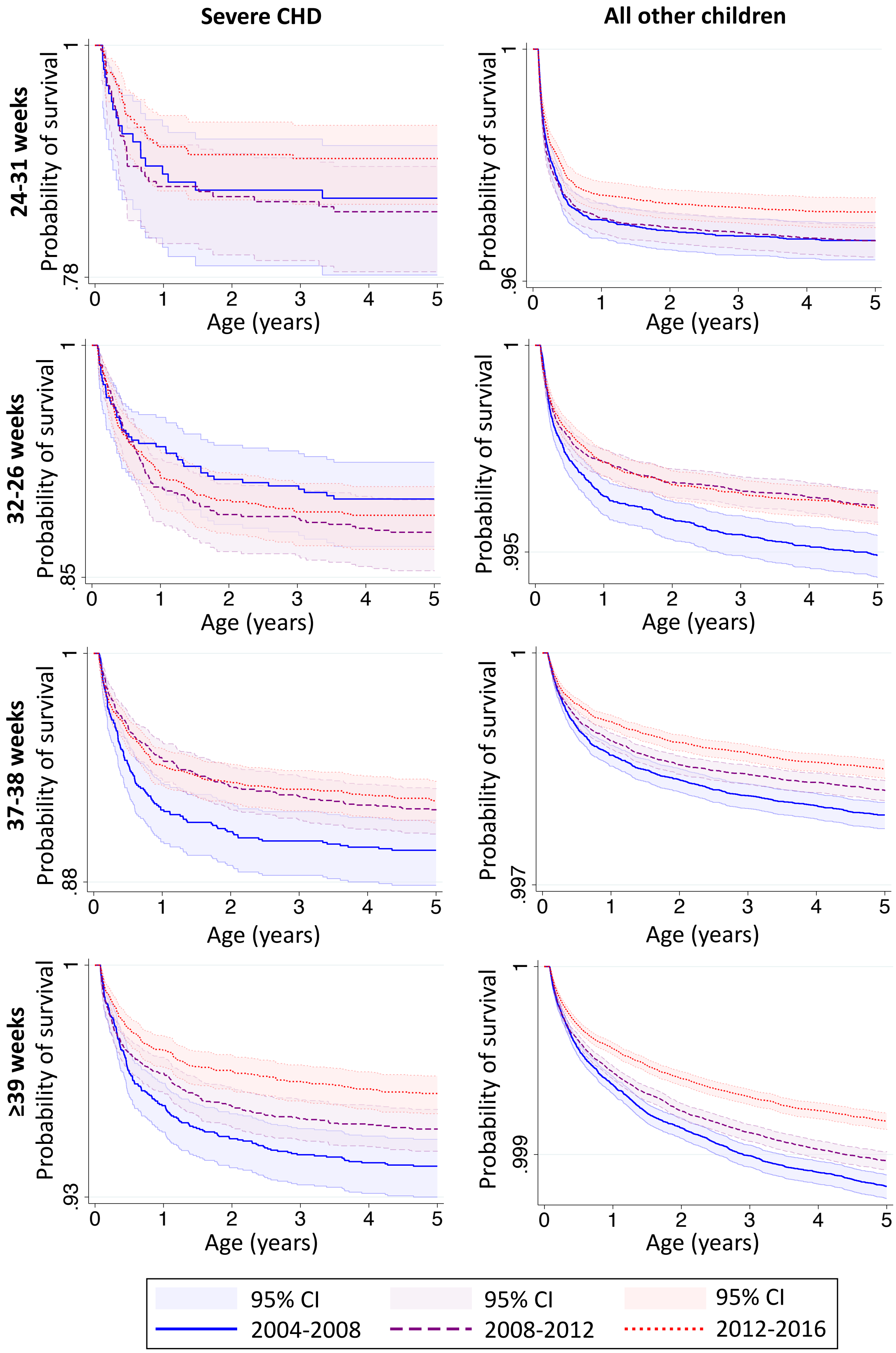

eFigure 5. Kaplan-Meier survival plots for children with severe CHD and all other children up to age five by gestational age-group

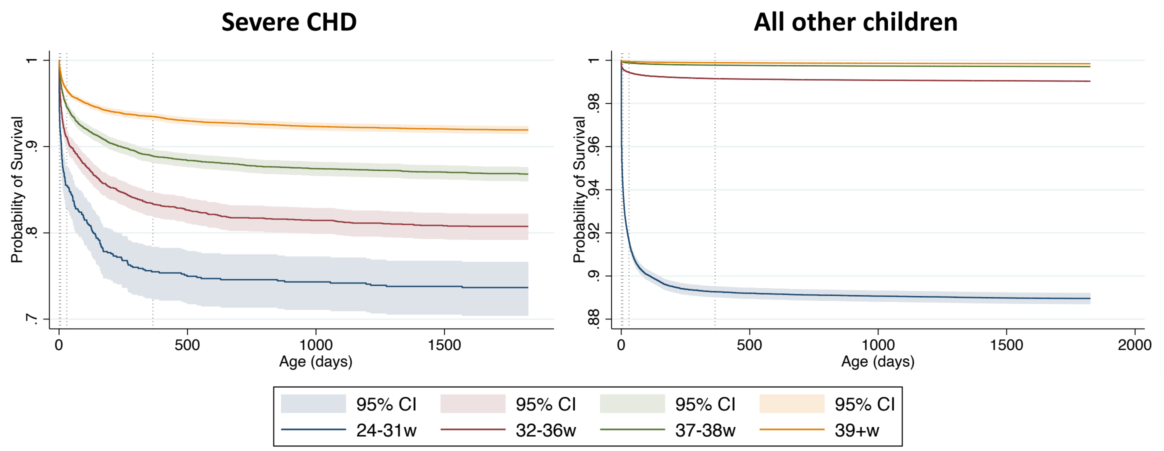

eFigure 6. Innovations in the care and management of children born preterm and with congenital heart defects since the 1960s. Innovations targeted at children with CHD are shown in italics.

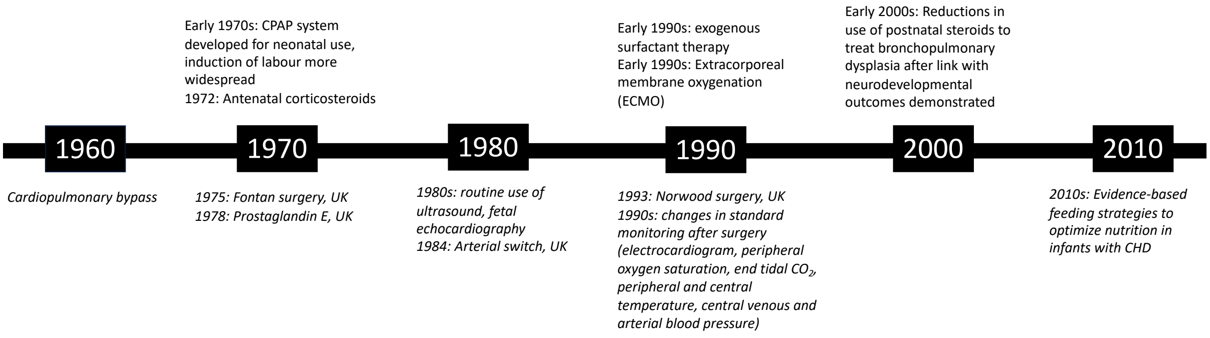

Supplement: Supplementary file 1 — Appendix S1. [file PPE-37-390-s001.pdf]
